# Dried blood spot as alternative specimen for molecular epidemiology studies among HCV/HIV coinfected patients

**DOI:** 10.1016/j.bjid.2025.104512

**Published:** 2025-02-21

**Authors:** Geane Flores, Barbara Vieira Lago, Amanda R Caetano, Jessica Silva, Vanessa Marques, Carlos Eduardo Brandão-Mello, Marcia Amendola-Pires, Jose Pilotto, Lia Lewis-Ximenez, Livia Melo Villar

**Affiliations:** a Instituto Oswaldo Cruz (Fiocruz), Laboratório de Hepatites Virais, Rio de Janeiro, RJ, Brazil; b Instituto de Tecnologia Imunobiológica (Biomanguinhos), Rio de Janeiro, RJ, Brazil; c Universidade Federal do Rio de Janeiro State, Hospital Gaffree and Guinle, Rio de Janeiro, RJ, Brazil; d Instituto Oswaldo Cruz (Fiocruz), Laboratório de AIDS e Imunologia Molecular, Rio de Janeiro, RJ, Brazil

**Keywords:** HIV, HCV, HIV/HCV co-infection, DBS

## Abstract

**Background:**

Immunodeficiency Virus (HIV) and Hepatitis C Virus (HCV) share the same routes of transmission, therefore, co-infection by both viruses represents a challenge to the goal of eliminating viral hepatitis as a public health threat. There are an estimated 2.3 million people living with HIV/HCV worldwide. Most of these cases affect vulnerable populations located in places with low infrastructure. Because of this, the use of alternative samples such as Dried Blood on Spot (DBS) would facilitate access to diagnosis and HCV treatment. The aim of this study is to evaluate the HCV genetic variability in HIV/HCV individuals by correlating paired serum and DBS samples.

**Methods:**

A total of 14 HIV/HCV individuals, recruited from reference outpatient clinics in the city of Rio de Janeiro/Brazil, were included. From them, 64 % were man, mean of age 54±7. HCV RNA from both serum and DBS samples was RT-PCR amplified and sequenced with HCV NS5B-specific oligonucleotides. All positive samples were submitted to phylogenetic analysis.

**Results:**

Serum mean HCV load was 6.2 ± 0.5 log IU/mL. All patients presented undetectable HIV RNA. The distribution of HCV genotypes/subgenotypes was 1a (4/14); 1b (5/14); 3a (4/14); and 4d (1/14). Most paired serum and DBS samples showed concordant results (genetic distance: 0.0 to 0.16). One individual showed discordance in the subtypes between serum and DBS. Three individuals presented the 316 N Resistance Associated Mutation (RAS) in both serum and DBS.

**Conclusion:**

Our results demonstrate the applicability of DBS for HCV molecular tracking in HIV/HCV coinfected patients for viral genomic surveillance in key and vulnerable populations.

## Introduction

The World Health Organization has established a plan to eliminate viral hepatitis by 2030 by reducing mortality by 65 % and the global incidence of hepatitis B and C by 90 %.^1^ Hepatitis C is responsible for 72 million of chronic cases worldwide, with about 1.5 million new infections/year.^2^

Due to overlapped routes of transmission, Hepatitis C virus (HCV) and Human Immunodeficiency Virus (HIV) coinfection represents a challenge to the goal of eliminating viral hepatitis as a public health threat. It is estimated that 2.3 million people (6.2 %) of the estimated 37.7 million HIV-infected people have serological evidence of HCV infection.^3^ However, this prevalence varies greatly among different population groups, with the highest rates in key populations such as men who have sex with men and People Who Inject Drugs (PWID).^4^, ^5^, ^6^, ^7^ Globally, it is estimated that 17.8 % (10.8–24.8) of People Who Inject Drugs (PWID) are living with HIV, from them, 52.3 % (42.4–62.1) are HCV-antibody positive.^8^ Moreover, chronic liver disease is a major cause of morbidity and mortality among HIV carriers, once HIV accelerates the progression of fibrosis, especially in patients with severe immunodeficiency.^1^

Furthermore, despite the availability of a pan genotypic treatment for HCV, knowledge about the genetic variability of circulating HCV strains in HIV-HCV co-infected patients is of great relevance to molecular epidemiology studies. In this context, providing rapid, reliable and cost-effective diagnosis for coinfected individuals is a key point to achieve the WHO goal.

Dried Blood Spot (DBS) samples have been used for molecular detection of Hepatitis B Virus (HBV) and HCV.^9^, ^10^, ^11^, ^12^ DBS is less-invasive, easy to collect, stable at room temperature and could be an alternative to improve the access to HCV molecular diagnosis in areas of limited infrastructure and in hard-to-reach populations.^13^^,^^14^ Therefore, DBS may be useful in molecular epidemiology studies once it leads to greater a population adherence.

At our knowledge, the influence of HIV infection on the molecular detection of HCV in DBS is poorly studied. In this context, this study aims to evaluate the applicability of DBS to access HCV genetic diversity in HCV-HIV coinfected patients.

## Methods

### Study population

A total of 14 individuals co-infected with HIV and HCV (HIV/HCV+) donated serum and DBS samples at the same time. These samples were part of biorepository of Viral Hepatitis Laboratory and were collected from 2013 to 2017. This study was approved by the Ethics Committee of Oswaldo Cruz Foundation. Written informed consent was obtained from each subject before sample collection.

### Sample collection

Paired serum and DBS samples were obtained, where 6 mL of whole blood was collected from each patient and 75 µL was placed on a Whatman 903 protein protection card (Whatman, GE Healthcare, NJ), until pre-set circular paper discs −12 mm forms were completely filled.

### HCV RNA detection in serum and DBS samples

For serum, HCV RNA was extracted using the QIAamp Viral RNA Mini Kit (Qiagen, Germany) according to the supplier's instructions. For the DBS samples, 3 discs (3 mm each) of DBS were used and the recommendations determined by the manufacturers. For DBS, 30 microliters of water was used for RNA elution.

### HCV and HIV RNA quantification

Anti-HCV serum reactive samples were submitted to real time PCR (Cobas Taqman HCV 2.0, Roche, USA). HIV viral load was determined using real time PCR Abbott, EUA respectively.

### Detection of HCV RNA in the NS5B region in serum and DBS samples

HCV RNA detection was performed using an RT nested PCR to amplify the HCV NS5B region using primers described by Sandres-Sauné et al.^15^ For DBS samples, cDNA volumes of 5 µL were evaluated.

### Sequence analysis

Sequence analysis of RNA obtained from DBS and serum samples was performed using PCR products after purification using a QIAquick gel extraction kit (QIAGEN, Hilden, Germany). Direct nucleotide sequencing was done in both directions using a Big Dye Terminator kit (version 3.1, Applied Biosystems, Foster City, CA, USA) and external and internal primers 15. Sequencing reactions were analyzed on an ABI3730 automated sequencer (Applied Biosystems). The sequencing protocol was performed as described by Otto et al.^16^ MEGA 7.0 software was used to align and analyze nucleotide sequences and to construct a phylogenetic tree using the maximum likelihood method under the General Time Reversible model (GTR) as the best-fit model. The reliability of the phylogenetic tree was evaluated by bootstrap test (1000 replicates). Consensus sequences from each HCV isolate (serum and DBS) were submitted to a web-based computer program for subtyping and prediction of phenotypic resistance mutations in the NS5B gene (Max-Planck Institut für Informatik, Alemanha, http://hbv.geno2pheno.org/index.php).

### Statistical analysis

Descriptive statistical analysis was performed by calculating the mean and standard deviation. The influence of laboratory parameters on the probability of obtaining an HCV sequence was assessed using an unpaired *t*-test with Welch correction for continuous variables and Fisher's exact test for categorical variables. Statistical analysis was performed using GraphPad InStat software.

## Results

### Demographic and epidemiological features

Serum and DBS samples from 14 subjects were evaluated. Most subjects were male (9/14; 64.2 %) and the mean age was 54 ± 7 years. Regarding the HCV-HIV transmission routes, 7/14 (50 %) subjects declared not knowing the source of (co)infection; 4/14 (28.6 %) subjects declared possible sexual transmission and 3/14 (21.4 %) declared horizontal transmission by blood transfusion. Although most individuals were unaware of the source of infection, risk factors were observed in all individuals, pointing to the vulnerabilities existing in this group. As shown in Table 1, 12/14 (85.7 %) presented more than one risk factor for HCV-HIV coinfection. Risk factors related to sexual exposure were observed in 11/14 (78.6 %) subjects. Although no participant declared drug use as a probable transmission route, this risk factor was observed in 5/14 (35.7 %) individuals (Table 1).

**Table 1:** Demographic, epidemiological and virological characteristics of HCV-HIV subjects.

Identification	Gender	Age (years)	Risk factors	Probable transmission route^a^	HCV viral load (log UI/mL)	HCV Genotype (serum/DBS)	HCV therapy
TSO1424	F	38	ICU, BT, S	Blood transfusion	6.49	1a/1a	IFN+RIB
TSO1863	F	45	ICU, MSP,S, PWUD, ISP	Unknown	5.22	1a/1a	No treatment
TSO1876	M	62	MSP	Unknown	5.92	1b/1b	INF
TSO1886	M	61	BT, S, ISP	Sexual	5.93	1b/1b	No information
TSO1887	M	55	ICU, S, PWUD	Unknown	6.75	1a/1a	No information
TSO1889	M	51	MSP, S, ISP	Unknown	6.05	3a/3a	No information
TSO1921	M	64	ICU, S	Sexual	6.78	1b/1a	IFN+RIBA
TSO1926	F	52	BT, S, ISP	Sexual	6.49	1b/1b	No information
TSO2041	M	54	MSP, BT, PWUD	Blood transfusion	5.82	3a/3a	IFN+Ribavirina
TSO2097	F	60	BT	Blood transfusion	6.19	1b/1b	IFN PEG + RBV
TSO2132	F	52	ISP	Sexual	6.6	3a/3a	No treatment
TSO2150	M	51	ICU, MSP	Unknown	7.06	4d	No information
TSO2179	M	60	S, PWUD	Unknown	5.61	3a/3a	No information
TSO2181	M	55	S, PWUD	Unknown	6.37	1a/1a	No information

a Self-declared. M, Male; F, Female; Y, Years; ICU, Irregular Condom Use; MSP, Multiple Sexual Partners; BT, Blood Transfusion; S, Surgery; PWUD, People Who Use Drugs; ISP, Infected Sexual Partner (HIV and/or HCV); DBS, Dried blood on a spot.

All subjects reported being under HIV therapy, however, only five patients had previous treatment for HCV (four patients were treated with interferon and ribavirin and one with interferon alone). Demographic, virological, and epidemiological characteristics are stated in Table 1.

### Molecular tests and genotyping

The mean HCV RNA viral load in serum was 6.2 ± 0.5 log IU /mL, and the genotype/ subgenotype distribution was: 1a (4/14); 1b (5/14); 3a (4/14); and 4d (1/14). In patients with genotype 1a, the mean HCV viral load was 6.20 ± 0.46 log IU/mL; among patients with genotype 1b was 6.20 ± 0.67 log IU/mL; patients with genotype 3 was 6.02 ± 0.42 log IU/mL and the patient infected with genotype 4 had a viral load of 7.06 log IU/mL. All individuals presented undetectable HIV loads.

All 14 paired serum and DBS samples were submitted to NS5B (partial) gene sequence analysis. A comparison between paired DBS and serum sequences displayed 0.0 to 0.16 genetic distance between them. One individual (TSO1921) showed discordance in the subtypes between serum and DBS (genetic distance: 0.16), being identified as subtype 1b in serum and 1a in DBS.

**Fig. 1:**
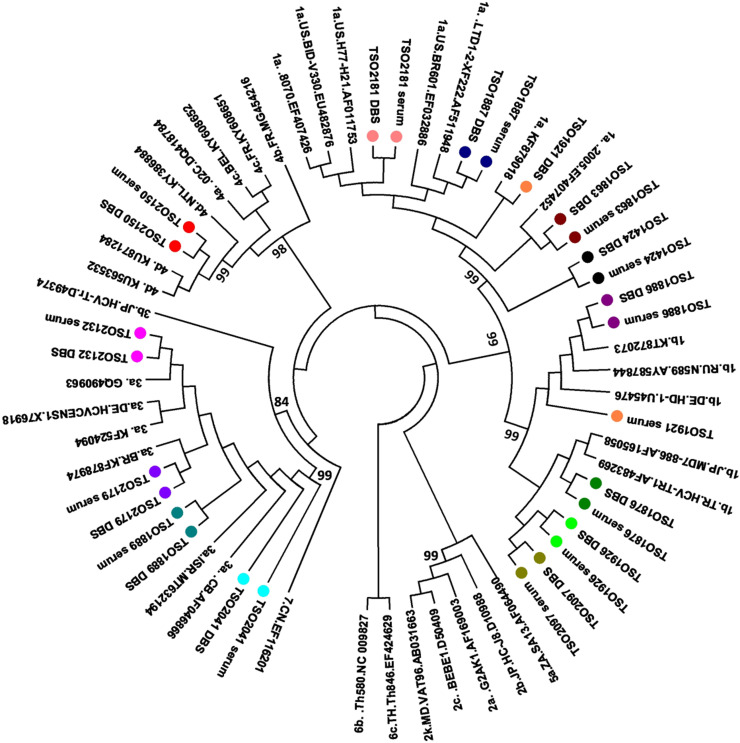
HCV phylogenetic tree (partial NS5B) performed by maximum likelihood method under the General Time Reversible model (GTR), bootstrap 1000 replicates. Serum and Dried blood on spot (DBS) pairs are stated in the same colors.

According to phylogenetic tree (Fig. 1), all genotypes 1 and 3 clustered with previously described Brazilian sequences,^17^^,^^18^ while sequence from genotype 4d clustered with sequences from The Netherlands.^19^ Based on the fragment available, no transmission cluster was observed among the study individuals.

Regarding Resistance Associated mutations (RAS), three individuals (TSO1876, TSO1926 and TSO2097) presented the 316 N mutation in both serum and DBS.

## Discussion

Although 18.9 % of people living with HIV in Brazil are currently coinfected with HCV, limited data is available on the molecular epidemiology and vulnerabilities faced by these individuals.^20^^,^^21^ In this study, we analyzed paired serum and DBS samples of 14 HCV-HIV coinfected patients, addressing risk factors and the HCV molecular features in both fluids. As observed by other authors,^21^^,^^22^ in this study, sexual exposure was the most common transmission route among HCV-HIV coinfected individuals. This can be attributed to the socio-behavioral vulnerabilities to which these individuals are exposed, such as irregular condom use, multiple sexual partners or sex with a partner who faces the same vulnerabilities.

Notably, we demonstrated the applicability of DBS to access HCV genotypes, RAS and genetic diversity in HCV-HIV coinfected patients. As demonstrated in previous studies, DBS sampling is a safe, less invasive, and cost-effective alternative to serum that may be used for molecular surveillance of infectious diseases.^9^, ^10^, ^11^^,^^23^ In this study, 100 % agreement in the genotype classification between paired DBS and serum samples was observed. In addition, three individuals had the RAS 316 N successfully detected in both fluids, further demonstrating the accuracy of DBS in detecting RAS. This substitution has been associated with an intermediate level of resistance to NS5B inhibitors and its early detection may impact future Direct-Acting Drug (DAA) treatments.^24^^,^^25^ Regarding subgenotypes, 93 % agreement was achieved, i.e., one individual in our study (TSO1921) showed discordant subgenotypes between serum and DBS (genetic distance: 0.16). Although incorrect nucleotide incorporation during PCR or sequencing cannot be ruled out, it is known that viral compartmentalization in distinct cellular reservoirs may play a role in viral *quasispecies* selection.^12^^,^^26^^,^^27^ TSO1921 is a 64-year-old MSM, under HCV therapy since 2004, who reported irregular condom use, history of STIs and who declared to have acquired HCV-HIV coinfection through sexual exposure. Taken these vulnerabilities together, it is reasonable to hypothesize that multiple viral introductions over time, coupled with pharmacological and host selective pressure might result in the coexistence of distinct subgenotypes in serum and peripheral Blood Mononuclear Cells (PBMC), where these *quasispecies* would be virtually more protected from selective pressure.

The distribution of HCV genotypes in our study is in agreement with other studies performed in Brazil.^28^, ^29^, ^30^ Here, genotypes 1a and 1b were the most prevalent (62 %), followed by genotype 3a (21 %) and genotype 4d (7 %). Similar findings were recently reported by Ferrufino and colleagues studying HIV/HCV co-infected individuals in Brazil, where genotypes 1 (49 %), 3 (19 %), 2 and 4 (1.5 %) were detected.^21^

Phylogenetic analysis revealed that genotypes 1 and 3 sequences clustered with previously described Brazilian samples,^17^^,^^18^ while genotype 4d was genetically related to sequences from The Netherlands circulating in MSM.^19^ Previous study had reported an increase in genotype 4 detection in HCV-HIV coinfected patients in Brazil from 2010 onward, possibly related to sexual exposure.^21^ This genotype has also been associated with HCV-HIV sexual transmission in European MSM.^19^^,^^31^^,^^32^ In our study, genotype 4d was found in the paired serum and DBS of a MSM who reported irregular condom use with multiple sexual partners, pointing to sexual exposure as a plausible route of infection. Similar finding was observed by Nutini and colleagues, who reported genotype 4 circulation in Brazilian HIV-HCV coinfected men.^30^ According to the authors, the sharp increase of the once rare genotype 4 in HCV-HIV coinfected men in recent years might be a result of founder effect and viral dispersion among members of a specific population, such as MSM.^30^ In this context, the accurate detection of genotype 4 in this and other studies has shown that DBS may be useful in identifying emerging viral genotypes.^33^^,^^34^

Although no transmission clusters were identified in this study, it is known that the lifestyle and socio-behavioral vulnerabilities to which HIV-HCV co-infected patients are continually exposed may lead to a high rate of HCV reinfection. Due to the need to provide continuous testing for this population, DBS sampling can be a strategy to expand diagnosis and promote genomic surveillance in places with low infrastructure and in hard-to-reach populations where blood collection, which is the gold standard for molecular epidemiology studies, is not possible or well accepted.

This study has some limitations, such as the limited number of paired samples, which is reflected in the restricted number of HCV genomes/genotypes analyzed. In addition, memory bias and the possibility of participants omitting risky practices in their self-report are also limitations of this study. Furthermore, it is important to note that the extraction of DBS for molecular analysis requires additional incubation steps which, taken together, take around 60 mins longer than serum extraction. Despite this increase in extraction time, this difference does not lead to a significant delay in the results.

## Conclusion

In conclusion, this study assessed HCV genetic variability in HIV co-infected patients comparing paired serum and DBS samples. Our results demonstrate that DBS is an accurate, cost-effective and non-invasive tool for epidemiologic molecular surveillance of viral variants in key and vulnerable populations. In addition, we point to the need to better understand the vulnerabilities associated with HCV infection routes in key populations, to address effective interventions.

## Statement of ethics

Study approval statement: The study was conducted in accordance with the Declaration of Helsinki, and approved by the Ethics Committee of Oswaldo Cruz Foundation.

Consent to participate statement: Written informed consent was obtained from all subjects involved in the study before sample collection.

## Authors’ contributions

All authors have read and agreed to the published version of the manuscript.

## CRediT authorship contribution statement

**Geane Flores:** Conceptualization, Formal analysis, Writing – original draft. **Barbara Vieira Lago:** Validation, Formal analysis, Writing – original draft, Writing – review & editing. **Amanda R Caetano:** Methodology, Formal analysis, Investigation. **Jessica Silva:** Methodology, Formal analysis, Investigation. **Vanessa Marques:** Methodology, Validation. **Carlos Eduardo Brandão-Mello:** Writing – review & editing, Visualization. **Marcia Amendola-Pires:** Writing – review & editing, Visualization. **Jose Pilotto:** Validation, Investigation. **Lia Lewis-Ximenez:** Resources, Supervision, Funding acquisition. **Livia Melo Villar:** Conceptualization, Resources, Writing – review & editing, Supervision, Funding acquisition.

## Conflicts of interest

The authors declare no conflicts of interest.
